# PET Demonstrates Functional Recovery after Treatment by Danhong Injection in a Rat Model of Cerebral Ischemic-Reperfusion Injury

**DOI:** 10.1155/2014/430757

**Published:** 2014-02-23

**Authors:** Zefeng Wang, Fahuan Song, Jinhui Li, Yuyan Zhang, Yu He, Jiehong Yang, Huifen Zhou, Tao Zhao, Wei Fu, Panke Xing, Haitong Wan, Mei Tian, Hong Zhang

**Affiliations:** ^1^Institute of Cardio-Cerebrovascular Diseases, Zhejiang Chinese Medical University, 548 Binwen Road, Hangzhou, Zhejiang 310053, China; ^2^Department of Nuclear Medicine, The Second Affiliated Hospital of Zhejiang University School of Medicine, 88 Jiefang Road, Hangzhou, Zhejiang 310009, China; ^3^Zhejiang University Medical PET Center, Zhejiang University, Hangzhou 310009, China; ^4^Key Laboratory of Medical Molecular Imaging of Zhejiang Province, Hangzhou 310009, China; ^5^Buchang Pharmaceutical Co., Ltd., Xi'an 712000, China

## Abstract

This study aimed to investigate neuroprotection of Danhong injection (DHI) in a rat model of cerebral ischemia using ^18^F-fluorodeoxyglucose positron emission tomography (^18^F-FDG-PET). *Method*. Rats were divided into 5 groups: sham group, ischemia-reperfusion untreated (IRU) group, DHI-1 group (DHI 1 mL/kg/d), DHI-2 group (DHI 2 mL/kg/d), and DHI-4 group (DHI 4 mL/kg/d). AII the treated groups were intraperitoneally injected with DHI daily for 14 days. The therapeutic effects in terms of cerebral infarct volume, neurological function, and cerebral glucose metabolism were evaluated. Expression of TNF-*α* and IL-1*β* was detected with enzyme-linked immunosorbent assay (ELISA). Levels of mature neuronal marker (NeuN), glial marker (GFAP), vascular density factor (vWF), and glucose transporter 1 (GLUT1) were assessed by immunohistochemistry. *Results*. Compared with the IRU group, rats treated with DHI showed dose dependent reductions in cerebral infarct volume and levels of proinflammatory cytokines, improvement of neurological function, and recovery of cerebral glucose metabolism. Meanwhile, the significantly increased numbers of neurons, gliocytes, and vessels and the recovery of glucose utilization were found in the peri-infarct region after DHI treatment using immunohistochemical analysis. *Conclusion*. This study demonstrated the metabolic recovery after DHI treatment by micro-PET imaging with ^18^F-FDG and the neuroprotective effects of DHI in a rat model of cerebral ischemic-reperfusion injury.

## 1. Introduction

Ischemic stroke is a leading cause of death, disability, and massive socioeconomic loss worldwide [1]. Current therapeutic strategies mainly focusing on restoring blood flow to the cerebral ischemia timely are frequently used in clinic [2]. But reperfusion after cerebral ischemia usually leads irreversible brain damage, which is driven by diverse pathological factors including inflammation, excitotoxicity, free radical-induced neuronal damage, and apoptosis [3–7]. There is no effective treatment for cerebral ischemic-reperfusion injury. Thus, traditional Chinese medicine (TCM) is focused on for its multitargets therapy. TCM has been used in ancient medical systems for treating various neurological diseases, especially stroke, and has exerted its distinctive neuroprotective effects on cerebral ischemia [8–10].

Danhong injection (DHI) is a standardized water-soluble product extracted from the Chinese herbal *Radix Salviae Miltiorrhizae* (Danshen) and *Flos Carthami* (Honghua) with modern technology. *Radix Salviae Miltiorrhizae* and *Flos Carthami* are both well-known Chinese herbal medicines and widely combined as a famous Chinese medicinal formula for treating cardiovascular and cerebrovascular diseases. According to the theory of TCM, the former is cold, while the latter is warm, so they are used together to open the blood vessels and promote blood flow in circulation [11]. DHI is composed of complex components, which is the central problem of quality stabilization and safety for clinic in past few decades. Based on this, its chromatographic fingerprint analysis has been investigated by high performance liquid chromatography coupled with diode-array detector (HPLC-DAD), and ten components of DHI were quantificationally identified as protocatechuic aldehyde, caffeic acid, danshensu, 5-hydroxymethyl-2-furfural, salvianolic acid D, salvianolic acid B, salvianolic acid A, lithospermic acid, ferulic acid, and rosmarinic acid [12].

DHI has been used in therapy for various diseases in animal experiment research or in clinic, including traumatic intracranial hematoma, hepatic venoocclusive disease, and myocardial reperfusion injury [11, 13, 14]. We previously have demonstrated the protective effect of DHI on cerebral ischemia reperfusion injury via anticoagulant, antithrombotic, antifibrinolytic, and antioxidant activities [15]. However, it is not enough for dissecting the underlying therapeutic mechanisms of DHI on ischemic stroke, as well as demanding more scientific and accurate evidence for their effectiveness and finding the principles behind them.

Molecular imaging is generally defined as the visual representation, characterization, and quantification of biological processes at the cellular and molecular levels within intact living organism [16]. Especially, positron emission tomography (PET) with ^18^F-fluorodeoxyglucose (^18^F-FDG) is a powerful tool to monitor the glucose metabolism quantitatively and noninvasively and has been widely used in assessment of the effects for cerebrovascular disease therapy [17, 18]. Hence, the present study mainly explored the effects of DHI on a rat mode induced by middle cerebral artery occlusion (MCAO) with ^18^F-FDG micro-PET evaluating the glucose metabolic recovery of the cerebral infarction area. Meanwhile, the activity of neurogenesis and angiogenesis on ischemic hemisphere was determined by histologic analysis to better understand the mechanisms of DHI therapy for cerebral ischemia reperfusion injury.

## 2. Materials and Methods

### 2.1. Animal and Experimental Design

Adult male Sprague-Dawley rats (body weight, 250–280 g) were purchased from the Animal Center of Zhejiang Chinese Medical University, Hangzhou, China (Laboratory Animal Certificate: scxk 2008-0115) and housed in the Key Laboratory of Medical Molecular Imaging of Zhejiang Province with a 12 h light-dark cycle, optimum temperature and humidity, filtrate water, and appropriate nutrient feed. All procedures related to care of animals were performed according to the National Institutes of Health Guide for the Care and Use of Laboratory Animals [19].

All rats were randomly divided into five experimental groups (15 per group): sham group, ischemia reperfusion untreated group (IRU), DHI-1 group (DHI, 1 mL/kg/d), DHI-2 group (DHI, 2 mL/kg/d), and DHI-4 group (DHI, 4 mL/kg/d). AII the treating groups were intraperitoneally injected with DHI daily for 14 days. PET imaging was performed at 1, 7, and 14 days after MCAO. Meanwhile, neurological functional tests were performed at 1, 3, 7, 10, and 14 days after MCAO. The rats were euthanized at 3, 7, and 14 days after MCAO for the determination of TNF-*α* and IL-1*β*, triphenyltetrazolium chloride assessment, and immunohistochemical detection, respectively.

### 2.2. Drug

DHI was produced by Shangdong Heze Buchang Pharmaceutical Co., Ltd. (Heze, China, Lot number 13011008), with a code number (Z20026866) approved by State Food and Drug Administration (SFDA) of China.

### 2.3. Induction of Middle Cerebral Artery Occlusion

All the rats were anesthetized with 1.5% pentobarbital sodium (50 mg/kg) and kept on the platform with a warm pad (model 69001; RWD Life Science, Shenzhen, China) to maintain the body temperature at 37°C throughout the whole procedure. Focal cerebral ischemia was induced by the intraluminal suture technology described by Longa et al. [20]. The right common carotid artery (CCA), the external carotid artery (ECA), and the internal carotid artery (ICA) were exposed through a midline skin incision in the neck. A 4-0 monofilament nylon suture with a rounded tip (Beijing Sunbio Biotech, Beijing, China) was carefully inserted into the ICA via ECA until a slight resistance was felt. After 90 min of MCAO, reperfusion was performed by slowly pulling the nylon suture back, and the wound was sutured. The rats of sham group were performed by the method of MCAO without inserting the nylon suture. All the animals were allowed to recover until being awake and were returned to their cages with optimum care.

### 2.4. Neurological Function Evaluation

Neurological function evaluations of each rat were performed at 1, 3, 7, 10, and 14 days after reperfusion of MCAO, as described previously [21]. Neurological defect was graded on a scale of 3–18 (3 = maximal deficit score; 18 = normal score). The score given to each rat is a composite of six individual test scores, including spontaneous activity, symmetry in the movement of the four limbs, forepaw outstretching, climbing, body proprioception, and response to touch of the vibrissae. To be brief, the lower the score, the more severe the injury.

### 2.5. TNF-*α* and IL-1*β* Determination

Rats (*n* = 5 for each group) were decapitated at 3 d after reperfusion and the brains were quickly taken out and stored at −80°C. The frozen ischemic brain tissues were weighed and homogenized. The homogenate was centrifuged at 3,000 g for 10 min and then the supernatants were collected to assay TNF-*α* (Cat number CK-E30419R) and IL-1*β* (Cat number CK-E30635R, Shanghai Yuanye Biological Technology, Shanghai, China) with enzyme-linked immunosorbent assay (ELISA) kits according to the manufacturers' instruction.

### 2.6. Infarct Volume Measurement

Rats (each group, *n* = 5) were euthanized by decapitation after 7 d of reperfusion. The brains were removed, then sliced into 2 mm coronal slices starting 1 mm from the frontal pole and immediately incubated in 2% 2,3,5-triphenyltetrazolium chloride (TTC, Sigma, St. Louis, USA) at 37°C for 15 min, and fixed in 4% paraformaldehyde overnight before analysis. Brain slices were scanned using a flat-bed scanner, and infarct volume was quantified with a computerized image analyzer. Infarct volume was expressed as a percentage of the total volume of slices [22].

### 2.7. In Vivo PET Study and Image Analysis

At 24 h, 7 d, and 14 d after reperfusion, rats were held still without anesthesia and injected with approximately 18.5 MBq of ^18^F-FDG via the tail vein. The rats were returned to their cage and were allowed to move freely for 60 min. Subsequently, the rats were anesthetized with 2% isoflurane and positioned with the mid skull in the center of micro-PET R4 scanner (Concorde Microsystems, Knoxville, USA). A ten min static acquisition was performed and the images were reconstructed using a maximum a posteriori probability algorithm. ^18^F-FDG uptake was calculated as the percentage of injected dose per gram (%ID/g) of tissue, using the ASIPro (Concorde Microsystems, Knoxville, USA). Regions of the interest (ROIs) were symmetrically manually drawn in the neocortex and striatum, and the ratio of ipsilateral to contralateral radioactivity was calculated. The right lesion to normal homologous contralateral (R/N) ratio was used for semiquantitative analysis.

### 2.8. Immunohistochemical Assessment

At 14 d after MCAO, the animals (5 per group) were deeply anesthetized with 1.5% pentobarbital sodium and transcardially perfused with saline followed by 4% paraformaldehyde dissolved in 0.1 M phosphate buffer. The brains were removed and fixed in fresh fixative for 6 h, followed by 30% sucrose in 0.1 M PBS overnight for dehydration. Then, the brains were dissected and 3 mm thick coronal sections were prepared for the following determination. For immunohistochemical analysis, the sections were treated with primary antibody against glial fibrillary acidic protein (GFAP, rabbit polyclonal antibody 1 : 300 dilution, Abcam Ab7260, USA), neuronal nuclei (NeuN, mouse monoclonal antibody, 1 : 200 dilution; clone: A60, Millipore MAB377, USA), von Willebrand factor (Vwf, rabbit polyclonal antibody, 1 : 200 dilution; DAKO GA008210, Denmark), or glucose transporter 1 (GLUT1; mouse monoclonal, 1 : 150 dilution, clone: 5B 12.3, Millipore MABS132, USA) at room temperature for 60 min. The sections were washed 3 times in PBS for 5 min each and incubated with secondary antiserum (EnVision Two-Step kit, DAKO 1701777A, Denmark) for 40 min at room temperature. Subsequently, the sections were washed 3 times with PBS and incubated with diaminobenzidine (DAB) and Hematoxylin staining solution (Harris) for 2-3 min until a brown reaction product was observed under a light microscope.

Immunohistochemical assessment was performed to determine whether DHI can promote neurogenesis and angiogenesis and the recovery of glucose utilization. NeuN, GFAP, and vWF were used as a mature neuronal marker, the mature astrocyte marker, and the endothelial cell marker, respectively. GLUT1 was used as a marker of glucose transporters localized in the endothelial cells. The number of cells positively stained with NeuN or vWF was counted in 3 different microscopic fields (351.1726 *μ*m × 468.5357 *μ*m, 200 magnification) and the mean values and SDs calculated. Furthermore, the integrated optical density (IOD) of GFAP and GLUT1 was achieved by averaging 3 microscopic fields (200 magnification) around the infarction area with use of Image-ProPlus 6.0 software (Media Cybernetics, Warrendale, USA).

### 2.9. Statistical Analysis

All data were expressed as mean ± SD, and multiple groups were compared by ANOVA. *P* values of less than 0.05 were considered statistically significant.

## 3. Results

### 3.1. Effects of DHI on Functional Recovery

**Figure 1:**
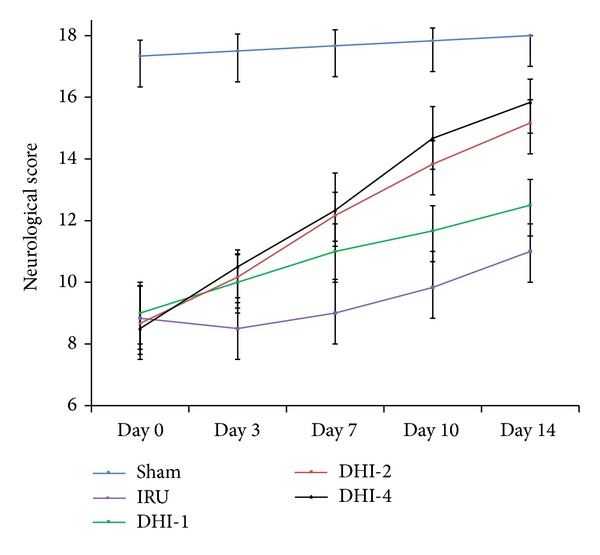
Neurological deficit score in ischemic rats after reperfusion. Sham group (Sham), ischemia-reperfusion untreated group (IRU), Danhong injection 1 mL/Kg group (DHI-1), Danhong injection 2 mL/Kg group (DHI-2), and Danhong injection 4 mL/Kg group (DHI-4) in Days 0, 3, 7, 10, and 14, respectively.

After MCAO, the neurological deficit score in the IRU group was significantly lower than that of the sham group (*P* < 0.05) on Days 0, 3, 7, 10, and 14. Meanwhile, the scores in the DHI-1 group (*P* < 0.05), DHI-2 group (*P* < 0.01), and DHI-4 group (*P* < 0.001), starting 3 d after reperfusion, were significantly higher at each time point than those of the IRU group. However, no significant differences in neurological function deficit scores were observed between the DHI-2 group and the DHI-4 group (*P* = 1.00). These results suggested that the treatment of DHI improved neurological function recovery (Table 1, Figure 1).

### 3.2. Effects of DHI on Infarct Volume

**Figure 2:**
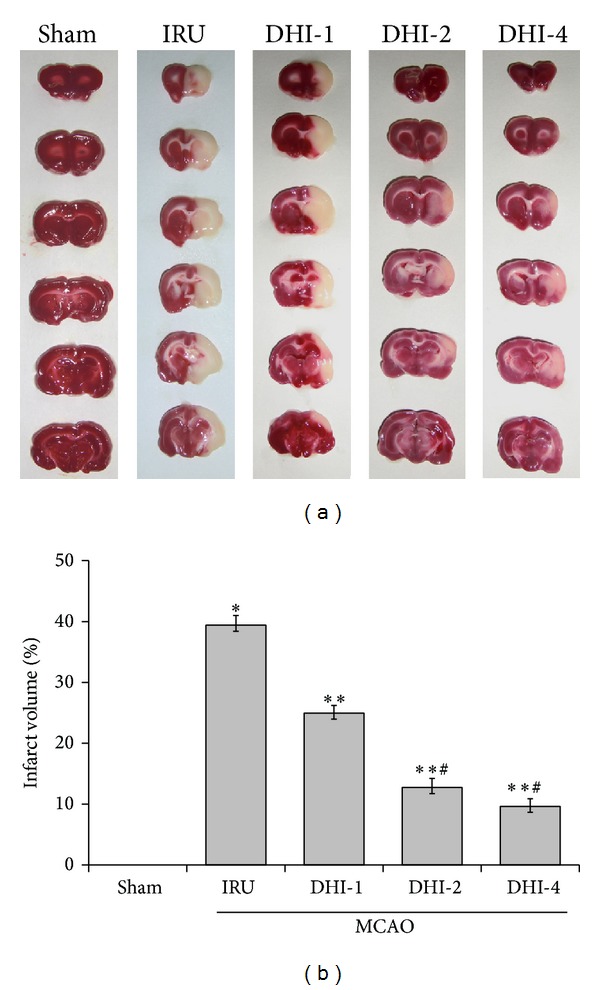
Infarct volume assessed by 2,3,5-triphenyltetrazolium chloride (TTC) staining 7 days after transient MCAO. (a) Representative TTC staining of the cerebral infarct in coronal sections of rat brain. (b) Infarct volumes assessed by TTC staining. Groups are as follows: Sham group (Sham), ischemia-reperfusion untreated group (IRU), Danhong injection 1 mL/Kg group (DHI-1), Danhong injection 2 mL/Kg group (DHI-2), and Danhong injection 4 mL/Kg group (DHI-4). The data are expressed as means ± SD (*n* = 5). *P < 0.01 versus Sham, **P < 0.01 versus IRU, ^#^ *P* < 0.01 versus DHI-1.

The infarct volume in the IRU group (39.40 ± 1.61%, *P* < 0.001) was significantly increased in comparison to the sham group (0.00 ± 0.00%). The DHI-4 group showed the smallest infarct volume (9.64 ± 1.24%, *P* < 0.001), followed by the DHI-2 group (12.73 ± 1.50%, *P* < 0.001) and the DHI-1 group (24.95 ± 1.24%, *P* < 0.01), when compared with the IRU group (Figure 2).

### 3.3. Effects of DHI on the Expression of TNF-*α* and IL-1*β* Levels

**Figure 3:**
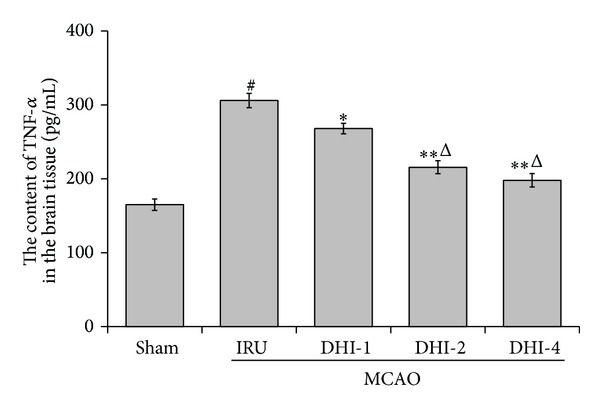
TNF-*α* expression detected by enzyme-linked immunosorbent assay in the brain tissue. Sham group (Sham), ischemia-reperfusion untreated group (IRU), Danhong injection 1 mL/Kg group (DHI-1), Danhong injection 2 mL/Kg group (DHI-2), and Danhong injection 4 mL/Kg group (DHI-4). The data are expressed as means ± SD (*n* = 5). ^#^ *P* < 0.01 versus Sham, *P < 0.05 versus IRU, **P < 0.01 versus IRU, and ^Δ^ *P* < 0.01 versus DHI-1.

**Figure 4:**
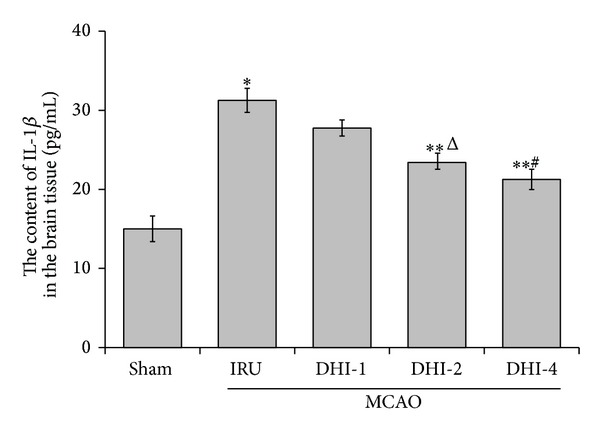
IL-1*β* expression detected by enzyme-linked immunosorbent assay in the brain tissue. Sham group (Sham), ischemia-reperfusion untreated group (IRU), Danhong injection 1 mL/Kg group (DHI-1), Danhong injection 2 mL/Kg group (DHI-2), and Danhong injection 4 mL/Kg group (DHI-4). The data are expressed as means ± SD (*n* = 5). *P < 0.01 versus Sham, **P < 0.01 versus IRU, ^Δ^ *P* < 0.05 versus DHI-1, and ^#^ *P* < 0.01 versus DHI-1.

In order to study whether the treatment of DHI can perform an anti-inflammatory pattern, we measured the expression of TNF-*α* and IL-1*β* levels in the brain tissue at 3 d after reperfusion by ELISA. The expression of TNF-*α* and IL-1*β* in the IRU group was significantly higher than that of the sham group (*P* < 0.001). The DHI-1 group, DHI-2 group, and DHI-4 group had significantly lower expression of TNF-*α* than the IRU group (*P* < 0.01). Meanwhile, the expression of IL-1*β* in the DHI-2 group (*P* < 0.05) and DHI-4 group (*P* < 0.01) was significantly decreased compared with the IRU group. Similarly, no significant difference was detected between the DHI-2 group and the DHI-4 group (Figures 3 and 4).

### 3.4. Effects of DHI on Glucose Metabolism

**Figure 5:**
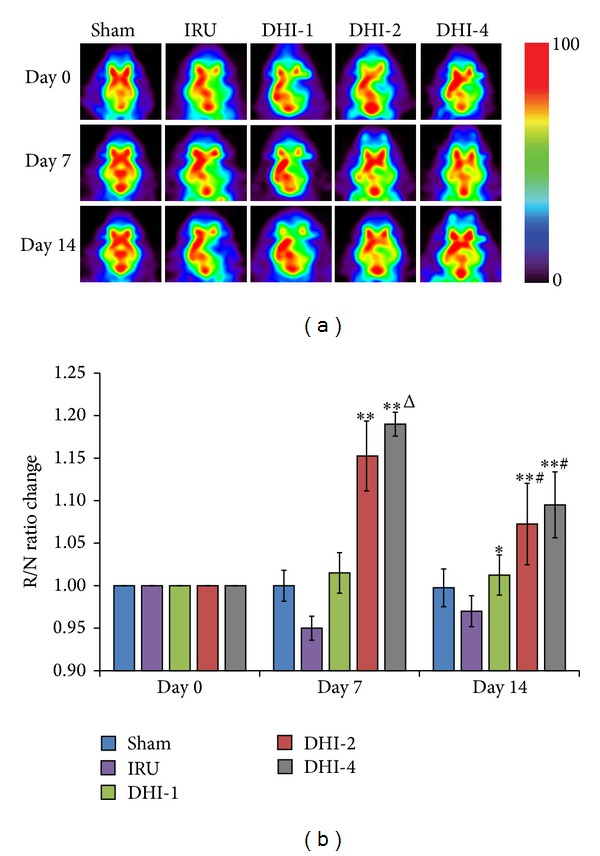
(a) ^18^FDG-PET images of activity of cerebral ischemic regions of the glucose metabolism. Sham group (Sham), ischemia-reperfusion untreated group (IRU), Danhong injection 1 mL/Kg group (DHI-1), Danhong injection 2 mL/Kg group (DHI-2), and Danhong injection 4 mL/Kg group (DHI-4). (b) Semiquantitative analysis of glucose metabolism after Danhong injection treatment in each group. The R/N ratio change was expressed as the R/N ratio at each time point after DHI-treatment relative to ratio of R/N before treatment. All the data are showed as means ± SD (*n* = 5). *P < 0.05 versus IRU, **P < 0.01 versus IRU, ^Δ^ *P* < 0.05 versus DHI-1, and ^#^ *P* < 0.01 versus DHI-1.

To study that the treatment of DHI corresponding to enhanced glucose metabolic activity, ^18^F-FDG PET scan was performed on the rats. With FDG-PET, the visualization and quantification of glucose metabolism of the brain was demonstrated at Day 0, Day 7, and Day 14 (Figure 5). Before DHI-treatment, the R/N ratio in the IRU group (0.66 ± 0.11, *P* < 0.001), the DHI-1 group (0.67 ± 0.10, *P* < 0.001), the DHI-2 group (0.73 ± 0.06, *P* < 0.001), and the DHI-4 group (0.62 ± 0.09, *P* < 0.01) were significantly lower than that in the Sham group (1.01 ± 0.01) and no significant difference was observed among them (*P* = 1.00). The R/N ratio change was expressed as the R/N ratio at each time point after DHI-treatment relative to ratio of R/N before treatment. After DHI-treatment, The R/N ratio change in the DHI-2 group and DHI-4 group both significantly increased compared with the IRU group at Day 7 and Day 14 (*P* < 0.01). For the DHI-1group, the R/N ratio change was only significantly higher than that in the IRU group at Day 14 (*P* < 0.05). However, there was no statistically significant difference between the DHI-2 treated group and the DHI-4 treated group in recovery of glucose metabolism at Day 7 and Day 14 (*P* = 1.00, *P* = 0.466, resp.).

### 3.5. Immunohistochemical Analysis

Immunohistochemical determination of NeuN, GFAP, and Vwf was performed to determine whether DHI can promote neurogenesis and angiogenesis. We counted the number of cells immunostaining positively with NeuN or vWF and calculated the mean values and SDs. In the ischemia reperfusion untreated animals, the number of NeuN-positive cells was significantly decreased compared with the Sham group (63.20 ± 6.42 versus 119.80 ± 8.93, *P* < 0.001). NeuN-positive cell number was significantly increased in the DHI-2 group (98.00 ± 8.09, *P* < 0.001) and DHI-4 group (102.40 ± 10.35, *P* < 0.001) in comparison with the IRU group. The DHI-2 group (*P* < 0.05) and DHI-4 group (*P* < 0.01) showed higher number of NeuN-positive cells than that in the DHI-1 group. The number of vWF-positive cells in both the DHI-4 group (15.60 ± 2.07, *P* < 0.01) and the DHI-2 group (14.00 ± 2.00, *P* < 0.05) was significantly higher than that in the IRU group (9.60 ± 1.52). The IRU group (*P* < 0.01) significantly increased the number of vWF-positive cells compared with the Sham group (5.60 ± 1.34). Furthermore, we measured IOD of GFAP and calculated the mean values and SDs. The IOD of GFAPs in the IRU group (23.01 ± 3.23, *P* < 0.001) significantly increased in comparison with the Sham group (4.55 ± 0.61). In the DHI-2 and DHI-4 treated animals, the IOD of GFAPs was significantly higher than that in the IRU group (33.49 ± 2.62 versus 4.55 ± 0.61, *P* < 0.001; 33.06 ± 2.81 versus 4.55 ± 0.61, *P* < 0.001, resp.). The DHI-2 group markedly increased the IOD of GFAPs compared with the DHI-1 group (33.49 ± 2.62 versus 27.87 ± 3.37, *P* < 0.05). However, there was no statistically significant difference between the DHI-2 treated group and the DHI-4 treated group in the expression of NeuN, GFAP, and vWF (*P* = 1.00).

Immunohistochemical study of GLUT1 was also performed to determine whether DHI can promote the recovery of glucose utilization. The IOD of GLUT1s in the IRU group was significantly higher than that in the Sham group (16.80 ± 1.89 versus 7.97 ± 1.52, *P* < 0.001). Meanwhile, the IOD of GLUT1s in the DHI-1 group (12.01 ± 2.67, *P* < 0.05), DHI-2 group (9.23 ± 2.22, *P* < 0.001), and DHI-4 group (7.68 ± 2.11, *P* < 0.001) markedly decreased in comparison with the IRU group. The DHI-4 group showed lower IOD of GLUT1s than that in the DHI-1 group (7.68 ± 2.11 versus 12.01 ± 2.67, *P* < 0.05). But no significant difference was observed between the DHI-2 and the DHI-4 group.

**Figure 6:**
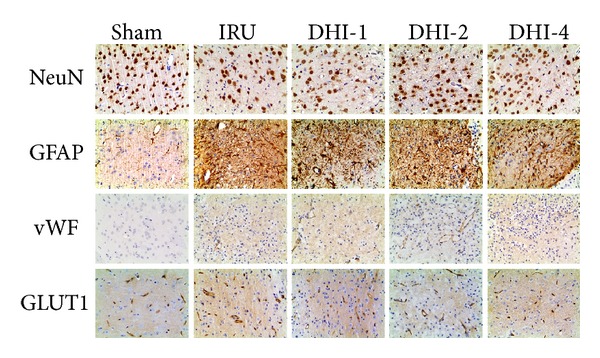
NeuN, GFAP, vWF, and GLUT1 immunostained tissue in ipsilateral peri-infarct region of focal cerebral ischemia in rats (magnification 200x). Sham group (Sham), ischemia-reperfusion untreated group (IRU), Danhong injection 1 mL/Kg group (DHI-1), Danhong injection 2 mL/Kg group (DHI-2), and Danhong injection 4 mL/Kg group (DHI-4).

**Figure 7:**
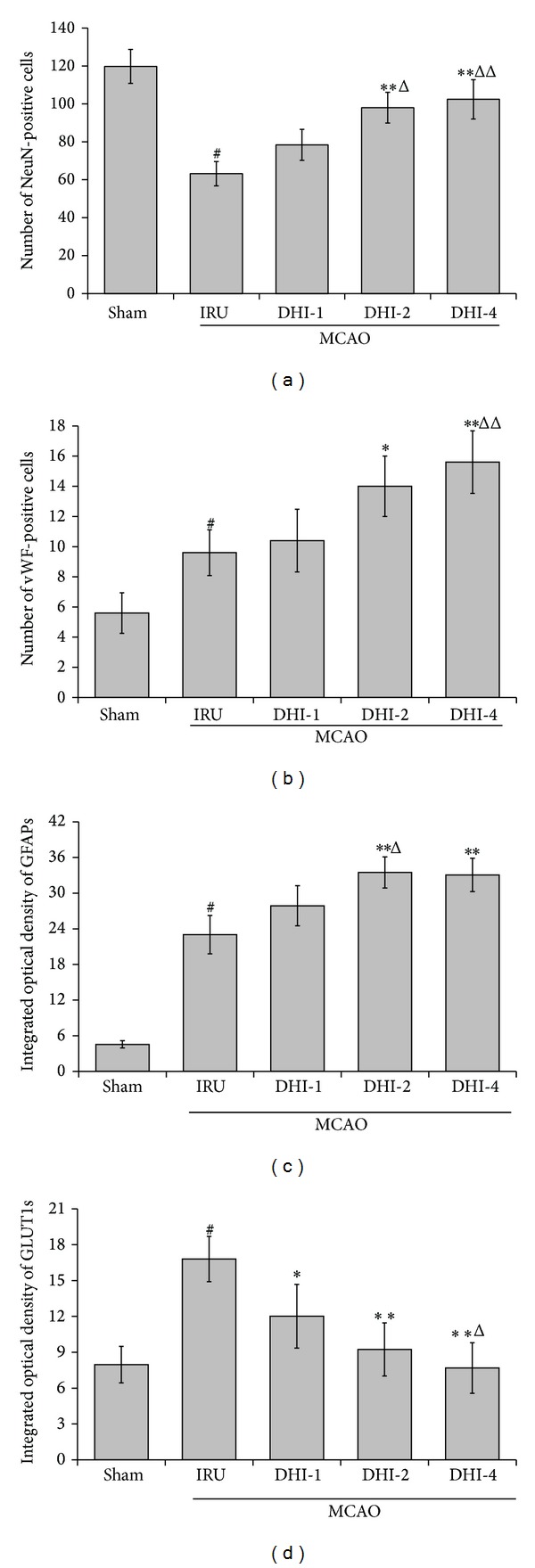
Immunohistochemical analysis of NeuN, GFAP, vWF, and GLUT1 in ipsilateral peri-infarct region. Sham group (Sham), ischemia-reperfusion untreated group (IRU), Danhong injection 1 mL/Kg group (DHI-1), Danhong injection 2 mL/Kg group (DHI-2), and Danhong injection 4 mL/Kg group (DHI-4). The data are expressed as means ± SD (*n* = 5). ^#^ *P* < 0.01 versus Sham, *P < 0.05 versus IRU, **P < 0.01 versus IRU, ^Δ^ *P* < 0.05 versus DHI-1, and ^ΔΔ^ *P* < 0.01 versus DHI-1.

All the data of immunochemistry analysis were summarized in Figures 6 and 7.

## 4. Discussion

In this study, the therapeutic effects of DHI were systematically evaluated by using a PET apparatus designed for small animals, combined with 4 other complementary approaches: neurological function evaluation, infarct volume assessment, immunohistochemical examination, and proinflammatory cytokines determination. And for all we know, it was the first time to use serial ^18^F-FDG PET to study the metabolic function recovery of MCAO-induced cerebral ischemia in rats after DHI treatment. During the 14-day period for DHI treatment, we found increased glucose metabolism in the area of cerebral infarction, decreased cerebral infarction volume, and improved neurological function by using a rat model of MCAO. Meanwhile, proinflammatory cytokines determination and immunohistochemical evaluation further dissected the potential therapeutic mechanisms of DHI treatment for anti-inflammation, promoting neurogenesis and angiogenesis, and promoting the recovery of glucose utilization.

In our previous study, we have used ^18^F-FDG PET to detect cerebral ischemia [17, 23, 24], since PET has the advantage of monitoring the glucose metabolism noninvasively and assessing the early effects for cerebrovascular disease therapy [25–27]. In the current study, ^18^F-FDG PET was used to evaluate cerebral glucose metabolic activity after the treatment of DHI. When compared with the IRU control group, we were able to find the higher ^18^F-FDG accumulation in the right cerebral infarction among the DHI treatment groups at Day 7 and Day 14 after MCAO, especially the DHI-2 group (*P* < 0.01) and the DHI-4 group (*P* < 0.01). The DHI-2 group and the DHI-4 group were better than the DHI-1 group in the recovery of glucose metabolism (Figure 5). Furthermore, data sampling was achieved under anesthesia with isoflurane in this study. However, the animals were performed the ^18^F-FDG injection without anesthesia and were allowed to move freely in their cage for 60 min after injection, suggesting that the uptake of ^18^F-FDG was essentially completed [28].

By assessment of neurological function deficits and infarct volume, we found that all of three DHI-treated groups improved neurological function recovery and reduced infarct volumes. Both the DHI-2 group and the DHI-4 group significantly increased the neurological score and reduced the cerebral infarct volume.

What are the mechanisms or factors that promote function recovery with DHI treatment after stroke? One possibility is that DHI treatment suppresses upregulation of proinflammatory cytokines TNF-*α* and IL-1*β*. Both TNF-*α* and IL-1*β* cytokines are supposed to play crucial roles in inflammatory cells infiltration and glia activation induced by cerebral ischemia, which exacerbate cerebral ischemia-reperfusion injury [29]. And previous studies have already proved that the injection of antagonists of TNF-*α* and IL-1*β* could relieve the ischemia injury [30, 31]. Our findings revealed that DHI treatment significantly suppressed the expression of proinflammatory cytokines TNF-*α* and IL-1*β* levels after ischemia. These changes indicate that anti-inflammatory activities play an important role during recovery.

In addition to its anti-inflammatory activities, the DHI treatment-induced neurogenesis and angiogenesis are crucial factors in functional recovery from cerebral ischemia. Recent studies present convincing evidence that endogenous neural stem cells exist in the subventricular zone (SVZ) of the lateral ventricle and dentate gyrus of the hippocampus in the mammalian adult brain [32], and ischemic stroke induced by MCAO triggers angiogenesis and promotes cell proliferation in the SVZ [33–35]. In addition, there is evidence that acupuncture and Chinese herb medicine, which are two important parts of TCM, enhance proliferation and differentiation of endogenous nerve stem cells in rats with focal cerebral ischemia [36–39]. In the present study, the number of vessels immunoreactive to vWF, an accepted marker for endothelial cells and angiogenesis [40], significantly increased in DHI-treated animals 14 days after MCAO compared with vehicle-treated ischemic animals. Furthermore, the expression of GFAP, a mature marker of astrocyte, significantly increased in DHI-treated groups compared with the IRU group. Following cerebral ischemia, the complex cascade of pathophysiological events occurred, ultimately leading to neuronal injury and death in brain ischemia [41]. It was confirmed by the significant decreased number of neurons immunoreactive to NeuN, a mature neuronal marker, in the IRU group compared with the Sham group in our study. After DHI treatment, the number of NeuN-positive cells was higher in the treatment groups than that in the vehicle-treated ischemic group. So we presume that the cerebral microvasculature multiple dynamic responses are evolved through microvascular propagation of ischemic and peri-ischemic areas, which nourish astrocyte and promote post-ischemic neurogenesis in DHI-treated groups [42].

Another reasonable explanation is that DHI treatment significantly prevents the pathologic upregulation of glucose transporter in the peri-infarct regions. Glucose transporters are the facilitative Na^+^-independent sugar transporters and have 13 isoforms [43]. GLUT1, one of the important glucose transporters, is localized in the endothelial cells in the brain [44]. In our study, the density of GLUT1 is markedly higher in the peri-infarct regions of the IRU group rats than the Sham group rats, suggesting the self-adaptation to ensure the glucose delivery to the tissue to protect the brain. However, the DHI treatment significantly suppressed its upregulation by improving glucose utilization in the peri-infarct regions, and a similar result was achieved by Miyamoto et al. [45]. However, the underlying mechanisms through which the DHI treatment suppresses the upregulation of GLUT1 is unclear, and further studies are necessary to elucidate this issue.

The present study has its limitations. On the one hand, we did not use immunohistochemical evaluation with double or triple staining of markers for neurogenesis and angiogenesis, which is better to show neurogenesis and angiogenesis in this study. On the other hand, we need to do a lot of work for the further study to provide the evidence to determine whether the neurogenesis comes from SVZ and explain why DHI can enhance neurogenesis to protect ischemic stroke.

## 5. Conclusion

This study demonstrated the metabolic recovery after DHI treatment by serial ^18^F-FDG micro-PET imaging and the neuroprotective effects of DHI in a rat model of cerebral ischemic-reperfusion injury.

## Figures and Tables

**Table 1:** Neurological deficit of five groups after cerebral ischemia/reperfusion in different days (mean ± SD).

Groups	*n*	Neurological score				
		Day 0	Day 3	Day 7	Day 10	Day 14
Sham	5	17.33 ± 0.52	17.50 ± 0.55	17.66 ± 0.52	17.83 ± 0.40	18.00 ± 0.00
IRU	5	8.83* ± 1.17	8.50* ± 0.84	9.00* ± 1.09	9.83* ± 1.16	11.00* ± 0.89
DHI-1	5	9.00* ± 0.89	10.00** ± 0.89	11.00** ± 0.89	11.67** ± 0.82	12.50** ± 0.84
DHI-2	5	8.66* ± 1.21	10.17*** ± 0.75	12.16*** ± 0.75	14.00*** ± 0.63	15.17*** ± 0.75
DHI-4	5	8.50* ± 1.37	10.50*** ± 0.54	12.33*** ± 1.21	14.66*** ± 1.03	15.83*** ± 0.75

*P < 0.01, significantly higher compared with values of sham group.

**P < 0.05, significantly lower compared with values of IRU group.

***P < 0.01, significantly lower compared with values of IRU group.
